# Supplementary material for: Research and Public Interest in Mindfulness in the COVID-19 and Post-COVID-19 Era: A Bibliometric and Google Trends Analysis
Source: Int J Environ Res Public Health. 2023 Feb 21;20(5):3807. doi: 10.3390/ijerph20053807 (PMC10000852; doi:10.3390/ijerph20053807)
Supplement: Supplementary file 1 [file ijerph-20-03807-s001.zip › ijerph-2197234-supplementary.pdf]

**Supplementary files**

**Table S1.** Formulas of trend lines adopted based on $R^2$ value for each search term.

| ($R^2$ value by trend line model) | Linear | 2nd-order polynomial | 3rd-order polynomial | Formula (y) of trend line adopted |
|---|---|---|---|---|
| **Mindfulness** | 0.8313* | 0.8437 | 0.9313 | 0.0014x - 53.534 |
| **Mental health** | 0.2513 | 0.8367* | 0.8379 | 3E-06$x^2$ - 0.2563x + 5298.2 |
| **Psychotherapy** | 0.2078 | 0.7929* | 0.8022 | 7E-07$x^2$ - 0.06x + 1275.9 |
| **Psychoactive substance** | 0.0047 | 0.4534* | 0.4542 | 1E-07$x^2$ - 0.0115x + 242.5 |
| **Antidepressants** | 0.3140 | 0.6681* | 0.6706 | 4E-07$x^2$ - 0.029x + 606.17 |

Note. * indicates the adopted trend line model.

**Table S2.** Pearson's correlation between the five search terms (Mindfulness, Mental health, Psychotherapy, Psychoactive substance, and Antidepressants) for the entire period (2004.12. to 2022.11.)

| | | Mindfulness total | Mental health total | Psychotherapy total | Psychoactive substance total | Antidepressants total |
|---|---|---|---|---|---|---|
| Mindfulness total | Pearson Correlation | 1 | .412 | -.432 | .055 | .485 |
| | Sig. (2-tailed) | | .000 | .000 | .419 | .000 |
| | N | 216 | 216 | 216 | 216 | 216 |
| Mental health total | Pearson Correlation | | 1 | .453 | .697 | .834 |
| | Sig. (2-tailed) | | | .000 | .000 | .000 |
| | N | | 216 | 216 | 216 | 216 |
| Psychotherapy total | Pearson Correlation | | | 1 | .627 | .344 |
| | Sig. (2-tailed) | | | | .000 | .000 |
| | N | | | 216 | 216 | 216 |
| Psychoactive substance total | Pearson Correlation | | | | 1 | .571 |
| | Sig. (2-tailed) | | | | | .000 |
| | N | | | | 216 | 216 |
| Antidepressants total | Pearson Correlation | | | | | 1 |
| | Sig. (2-tailed) | | | | | |

| | | | | | |
|---|---|---|---|---|---|
| N | | | | | 216 |

**Period 1 (2004.12 to 2007.11)**

| | | Mindfulness P1 | Mental health P1 | Psychotherapy P1 | Psychoactive substance P1 | Antidepressants P1 |
|---|---|---|---|---|---|---|
| Mindfulness P1 | Pearson Correlation | 1 | .011 | -.142 | .069 | -.115 |
| | Sig. (2-tailed) | | .947 | .409 | .689 | .505 |
| | N | 36 | 36 | 36 | 36 | 36 |
| Mental health P1 | Pearson Correlation | | 1 | .739 | .800 | .790 |
| | Sig. (2-tailed) | | | **.000** | **.000** | **.000** |
| | N | | 36 | 36 | 36 | 36 |
| Psychotherapy P1 | Pearson Correlation | | | 1 | .677 | .791 |
| | Sig. (2-tailed) | | | | **.000** | **.000** |
| | N | | | 36 | 36 | 36 |
| Psychoactive substance P1 | Pearson Correlation | | | | 1 | .731 |
| | Sig. (2-tailed) | | | | | **.000** |
| | N | | | | 36 | 36 |
| Antidepressants P1 | Pearson Correlation | | | | | 1 |
| | Sig. (2-tailed) | | | | | |

| N | | | | | 36 |
|---|---|---|---|---|---|

**Period 2 (2007.12 to 2010.11)**

| | | Mindfulness P2 | Mental health P2 | Psychotherapy P2 | Psychoactive substance P2 | Antidepressants P2 |
|---|---|---|---|---|---|---|
| Mindfulness P2 | Pearson Correlation | 1 | -.030 | .120 | .332 | .430 |
| | Sig. (2-tailed) | | .862 | .487 | **.048** | **.009** |
| | N | 36 | 36 | 36 | 36 | 36 |
| Mental health P2 | Pearson Correlation | | 1 | .609 | .661 | .121 |
| | Sig. (2-tailed) | | | **.000** | **.000** | **.484** |
| | N | | 36 | 36 | 36 | 36 |
| Psychotherapy P2 | Pearson Correlation | | | 1 | .419 | .407 |
| | Sig. (2-tailed) | | | | **.011** | **.014** |
| | N | | | 36 | 36 | 36 |
| Psychoactive substance P2 | Pearson Correlation | | | | 1 | .253 |
| | Sig. (2-tailed) | | | | | .136 |
| | N | | | | 36 | 36 |
| Antidepressants P2 | Pearson Correlation | | | | | 1 |
| | Sig. (2-tailed) | | | | | |

| | | | | | |
|---|---|---|---|---|---|
| N | | | | | 36 |

**Period 3 (2010.12 to 2013.11)**

| | | Mindfulness P3 | Mental health P3 | Psychotherapy P3 | Psychoactive substance P3 | Antidepressants P3 |
|---|---|---|---|---|---|---|
| Mindfulness P3 | Pearson Correlation | 1 | .306 | -.033 | -.230 | -.214 |
| | Sig. (2-tailed) | | .069 | .850 | .177 | .210 |
| | N | 36 | 36 | 36 | 36 | 36 |
| Mental health P3 | Pearson Correlation | | 1 | .561 | .264 | .484 |
| | Sig. (2-tailed) | | | **.000** | .119 | **.003** |
| | N | | 36 | 36 | 36 | 36 |
| Psychotherapy P3 | Pearson Correlation | | | 1 | .540 | .354 |
| | Sig. (2-tailed) | | | | **.001** | **.034** |
| | N | | | 36 | 36 | 36 |
| Psychoactive substance P3 | Pearson Correlation | | | | 1 | .092 |
| | Sig. (2-tailed) | | | | | .594 |
| | N | | | | 36 | 36 |
| Antidepressants P3 | Pearson Correlation | | | | | 1 |
| | Sig. (2-tailed) | | | | | |

| N | | | | | 36 |
| --- | --- | --- | --- | --- | --- |

**Period 4 (2013.12 to 2016.11)**

| | | Mindfulness P4 | Mental health P4 | Psychotherapy P4 | Psychoactive substance P4 | Antidepressants P4 |
|---|---|---|---|---|---|---|
| Mindfulness P4 | Pearson Correlation | 1 | .695 | .541 | .223 | .527 |
| | Sig. (2-tailed) | | **.000** | **.001** | .190 | **.001** |
| | N | 36 | 36 | 36 | 36 | 36 |
| Mental health P4 | Pearson Correlation | | 1 | .590 | .418 | .487 |
| | Sig. (2-tailed) | | | **.000** | **.011** | **.003** |
| | N | | 36 | 36 | 36 | 36 |
| Psychotherapy P4 | Pearson Correlation | | | 1 | .357 | .512 |
| | Sig. (2-tailed) | | | | **.033** | **.001** |
| | N | | | 36 | 36 | 36 |
| Psychoactive substance P4 | Pearson Correlation | | | | 1 | .080 |
| | Sig. (2-tailed) | | | | | .642 |
| | N | | | | 36 | 36 |
| Antidepressants P4 | Pearson Correlation | | | | | 1 |
| | Sig. (2-tailed) | | | | | |

| | | | | | |
|---|---|---|---|---|---|
| N | | | | | 36 |

**Period 5 (2016.12 to 2019.11)**

| | | Mindfulness P5 | Mental health P5 | Psychotherapy P5 | Psychoactive substance P5 | Antidepressants P5 |
|---|---|---|---|---|---|---|
| Mindfulness P5 | Pearson Correlation | 1 | .781 | .739 | .559 | .688 |
| | Sig. (2-tailed) | | **.000** | **.000** | **.000** | **.000** |
| | N | 36 | 36 | 36 | 36 | 36 |
| Mental health P5 | Pearson Correlation | | 1 | .846 | .566 | .766 |
| | Sig. (2-tailed) | | | **.000** | **.000** | **.000** |
| | N | | 36 | 36 | 36 | 36 |
| Psychotherapy P5 | Pearson Correlation | | | 1 | .541 | .816 |
| | Sig. (2-tailed) | | | | **.001** | **.000** |
| | N | | | 36 | 36 | 36 |
| Psychoactive substance P5 | Pearson Correlation | | | | 1 | .582 |
| | Sig. (2-tailed) | | | | | **.000** |
| | N | | | | 36 | 36 |
| Antidepressants P5 | Pearson Correlation | | | | | 1 |
| | Sig. (2-tailed) | | | | | |

| | | | | | |
|---|---|---|---|---|---|
| N | | | | | 36 |

**Period 6 (2019.12 to 2022.11)**

| | | Mindfulness P6 | Mental health P6 | Psychotherapy P6 | Psychoactive substance P6 | Antidepressants P6 |
|---|---|---|---|---|---|---|
| Mindfulness P6 | Pearson Correlation | 1 | -.235 | -.056 | -.015 | -.470 |
| | Sig. (2-tailed) | | .167 | .745 | .931 | **.004** |
| | N | 36 | 36 | 36 | 36 | 36 |
| Mental health P6 | Pearson Correlation | | 1 | .578 | .498 | .731 |
| | Sig. (2-tailed) | | | **.000** | **.002** | **.000** |
| | N | | 36 | 36 | 36 | 36 |
| Psychotherapy P6 | Pearson Correlation | | | 1 | .331 | .725 |
| | Sig. (2-tailed) | | | | **.049** | **.000** |
| | N | | | 36 | 36 | 36 |
| Psychoactive substance P6 | Pearson Correlation | | | | 1 | .380 |
| | Sig. (2-tailed) | | | | | **.022** |
| | N | | | | 36 | 36 |
| Antidepressants P6 | Pearson Correlation | | | | | 1 |
| | Sig. (2-tailed) | | | | | |

| | | | | | |
|---|---|---|---|---|---|
| N | | | | | 36 |

**Figure S1.** Number of search results for MBCT on PubMed (from 1982 to 2022).

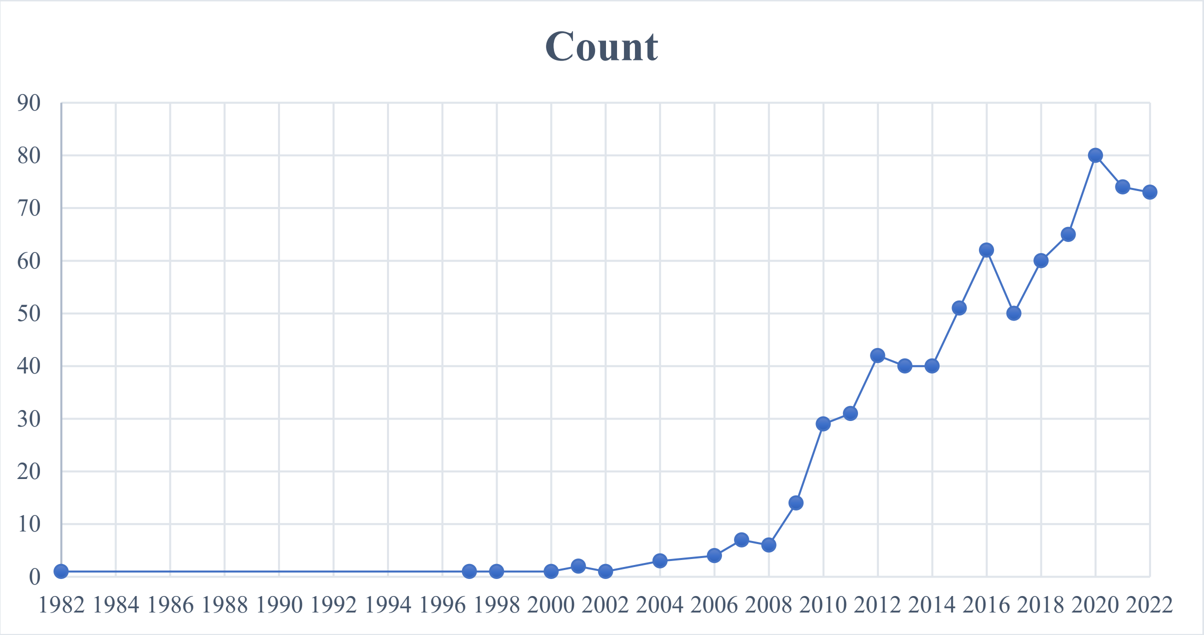

**Figure S2.** Relative search volume of MBCT in Google Trends (from 2004 to 2022, monthly).

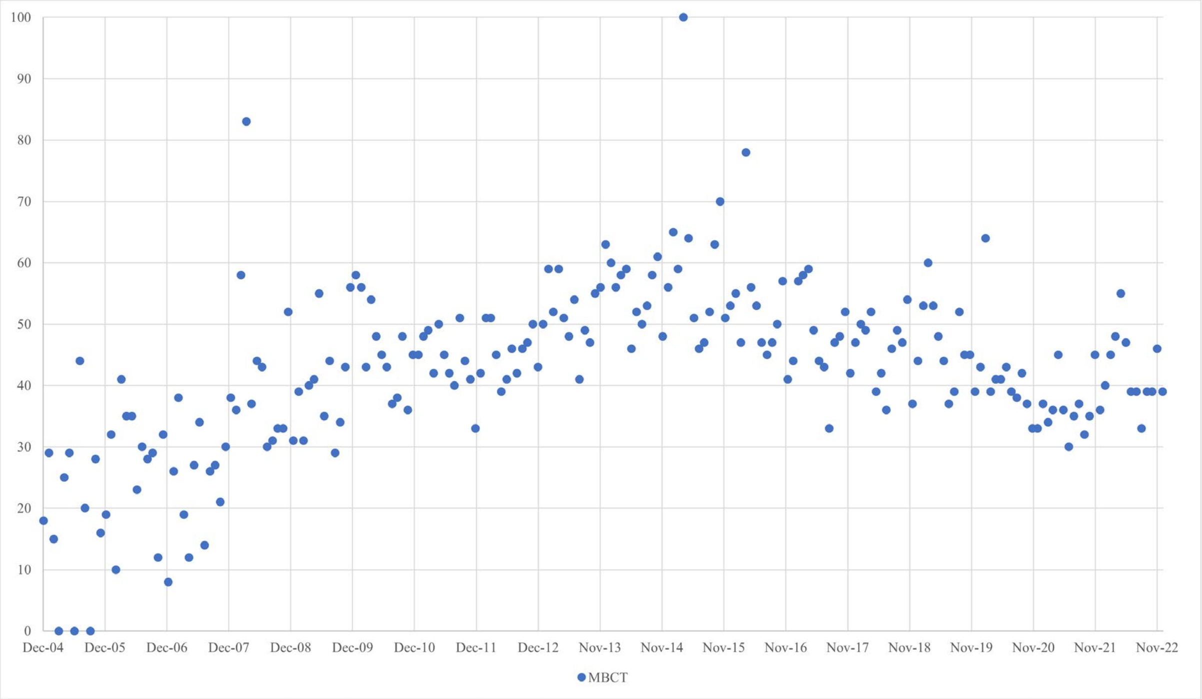


**Note**. The x-axis represents the relative search score and is distributed between 0 and 100 points. The y-axis represents the period.
